# Differentiation-Associated Reprogramming of the Transforming Growth Factor β Receptor Pathway Establishes the Circuitry for Epithelial Autocrine/Paracrine Repair

**DOI:** 10.1371/journal.pone.0051404

**Published:** 2012-12-19

**Authors:** Jonathan M. Fleming, Saqib Shabir, Claire L. Varley, Lisa A. Kirkwood, Angela White, Julie Holder, Ludwik K. Trejdosiewicz, Jennifer Southgate

**Affiliations:** 1 Jack Birch Unit for Molecular Carcinogenesis, Department of Biology, University of York, York, United Kingdom; 2 GlaxoSmithKline R&D, Ware, United Kingdom; University of Cincinnati, United States of America

## Abstract

Transforming growth factor (TGF) β has diverse and sometimes paradoxical effects on cell proliferation and differentiation, presumably reflecting a fundamental but incompletely-understood role in regulating tissue homeostasis. It is generally considered that downstream activity is modulated at the ligand:receptor axis, but microarray analysis of proliferative versus differentiating normal human bladder epithelial cell cultures identified unexpected transcriptional changes in key components of the canonical TGFβ R/activin signalling pathway associated with cytodifferentiation. Changes included upregulation of the transcriptional modulator SMAD3 and downregulation of inhibitory modulators SMURF2 and SMAD7. Functional analysis of the signalling pathway revealed that non-differentiated normal human urothelial cells responded in paracrine mode to TGFβ by growth inhibition, and that exogenous TGFβ inhibited rather than promoted differentiation. By contrast, in differentiated cell cultures, SMAD3 was activated upon scratch-wounding and was involved in promoting tissue repair. Exogenous TGFβ enhanced the repair and resulted in hyperplastic scarring, indicating a feedback loop implicit in an autocrine pathway. Thus, the machinery for autocrine activation of the SMAD3-mediated TGFβR pathway is established during urothelial differentiation, but signalling occurs only in response to a trigger, such as wounding. Our study demonstrates that the circuitry of the TGFβR pathway is defined transcriptionally within a tissue-specific differentiation programme. The findings provide evidence for re-evaluating the role of TGFβR signalling in epithelial homeostasis as an autocrine-regulated pathway that suppresses differentiation and promotes tissue repair. This provides a new paradigm to help unravel the apparently diverse and paradoxical effect of TGFβ signalling on cell proliferation and differentiation.

## Introduction

The high capacity of epithelial tissues for self-repair and renewal is effected through locally-regulated proliferation and differentiation of resident progenitor cells rather than recruitment of exogenous progenitor cells to the site. A dysfunction of regenerative mechanism(s) lies at the heart of numerous age-related diseases that afflict epithelial tissues, from chronic wounding and inflammation through to cancer, making epithelia a key target for regenerative and replacement therapies. As a model epithelium for regenerative studies, the uro-epithelial lining of the bladder and associated urinary tract provides an excellent system, being mitotically-quiescent with a low constitutive turnover rate, but with an exceptionally high capacity for regeneration [1]. In addition, urothelium expresses specific, highly objective markers of terminal differentiation, such as the uroplakins [2], [3], that define its specialised function as a urinary barrier.

In determining the mechanisms that orchestrate and mediate regeneration, a common perception is that the epithelium relies on the subjacent stroma. Indeed, an elegant recent study in the mouse bladder has provided evidence of an inductive paracrine loop operating between urothelium and stroma [4]. Nevertheless, there is also considerable evidence for autonomous growth regulation, for example through autocrine activation of epidermal growth factor receptor (EGFR) signalling [5].

The TGFβ superfamilies of ligands and cognate receptors act as major regulators of tissue development and homeostasis. Although TGFβ is thought of primarily as an anti-proliferative agent, it can also influence cell migration and promote apoptosis, and crucially, act as an inducer of epithelial to mesenchymal transition (EMT), a process implicated in tissue differentiation and wound-healing [6]. The precise effects of TGFβ can be paradoxical – for example, it may act as growth inhibitor or mitogen depending on concentration, cell type and context [7]. Similarly, its pro-apoptotic effects can be context-dependent, as illustrated by the observation that TGFβ promotes death by neglect only of post-activated T cells, but has no effect during T cell activation [8].

Responses to TGFβ are governed at several levels, including the nature of the ligand and the cell type-specific transcriptome that specifies the repertoire of surface receptors, the downstream SMAD-mediated and interactive signal transduction pathways and available transcriptional targets. In concert, these provide the basis for a cellular response that is influenced by the proliferative, differentiated and pathogenic status of the cell [9].

Much of the understanding of the role of TGFβ in human cells derives from studies of tumour-derived cell lines maintained in “simple” two-dimensional culture. In order to address its role in a more sophisticated tissue system, we have exploited a normal human epithelial cell culture system that we have previously demonstrated can be switched from a regenerative to differentiated phenotype. In serum-free, low calcium conditions, normal human urothelial (NHU) cells display a highly proliferative regenerative phenotype and do not express markers of urothelial differentiation, even at confluency [10]. These same cultures self-organise to form a stratified, functionally-differentiated urothelium when switched to appropriate in vitro conditions [11]. Alternatively, terminal differentiation may be induced pharmacologically by activation of PPARγ with concurrent inhibition of EGFR; this initiates a programme of transcriptional changes that results in expression of a late/terminal-differentiated urothelial cell phenotype [12], [13], [14].

In this study. we applied a microarray approach to identify gene expression changes common to both in vitro methods for inducing urothelial cytodifferentiation. The discovery of transcriptional changes in the canonical TGFβ signalling pathway suggested that TGFβ-related signalling was involved in the cytodifferentiation process. To test this hypothesis, the effects of exogenous and endogenous TGFβ signalling on cell growth, differentiation and wound repair were examined. Our data support a number of unexpected conclusions leading to a re-evaluation of the precise role of TGFβR signalling in epithelial homeostasis.

## Materials and Methods

### Materials

PD153035 (EGFR tyrosine kinase inhibitor; Calbiochem); SB431542 (TGFβ Superfamily Type I Activin Receptor-Like Kinase receptor inhibitor; Sigma) and troglitazone (TZ: PPARγ agonist; gift from Parke-Davis Pharmaceutical Research) were dissolved in DMSO. Human recombinant TGFβ1 (R&D Systems) and TGFβ2 (Peprotech) were dissolved in buffer. Appropriate solvent controls were included in all experiments.

Rabbit affinity-purified heteroantibodies against AKT, pAKT, pERK and pSMAD3 (Cell Signaling Technology), and against SMAD3 (Abcam) were used together with monoclonal antibodies to ERK (clone 16; Transduction Laboratories), Ki67 (clone MM1; Novocastra)), cyclin D1 (clone DCS-6; Cell Signaling Technology) and β-actin (clone AC-15; Sigma). Secondary antibodies used for western blot analysis were Alexa 680-conjugated goat anti-mouse IgG or Alexa 800-conjugated goat anti-rabbit IgG (Invitrogen) and for immunofluorescence were goat anti-mouse or goat anti-rabbit secondary antibody conjugated to Alexa 488 (green) or Alexa 594 (red) (Invitrogen).

### Cell culture

Human biological samples were sourced ethically with informed written consent from patients and approval for use in research from the Leeds (East) and the York Research Ethics Committees.

Finite NHU cell lines were established as detailed elsewhere [15], [16]. For routine propagation, cultures were maintained as monolayers in low calcium (0.09 mM) Keratinocyte Serum Free Medium (Invitrogen) containing bovine pituitary extract and EGF, and supplemented with cholera toxin (KSFMc). Cultures were sub-cultured at just-confluence as described [15], [16] and used for experiments between passages 3 to 5.

For induction of differentiation, NHU cell cultures were treated with either a) 1 µM TZ with concurrent 1 µM PD153035 to block downstream EGFR signalling (TZ/PD; [13]) or b) 5% adult bovine serum (ABS, Harlan Sera-Lab) and 2 mM CaCl_2_ (ABS/Ca^2+^) as described [11]). Control (non-differentiated) cultures were maintained in parallel in KSFMc and used at the same time points (between 24 to 144 hours). Cultures were lysed in situ with TRIzol® to prepare RNA by the manufacturer's recommended protocol (Invitrogen). RNA samples were treated with a DNA-free kit (Ambion) and quantified by UV spectrophotometry.

The HeLa-S3 cell line was used in some experiments as a positive control.

### UPK2-eGFP lentivirus

A lentivirus was produced using the ViralPower™ Promoterless Lentiviral Gateway® Expression system (Invitrogen) in which enhanced GFP (eGFP) was expressed under the control of the UPK2 promoter. The 1.8 kb functional fragment of the UPK2 promoter [17] was amplified from 500 ng of human genomic DNA using Expand high fidelity Taq polymerase enzyme (Roche) using primers 5′ AGG CTT CAC CCC AGA CCC ACT GC3′ and 5′GCT GGG CTG GGA GGT GGA ATA GG 3′. The resulting fragment was ligated into the pENTR™ 5′-TOPO® TA cloning vector using topoisomerase, transformed into TOP10 *E.coli* and selected in kanamycin (50 µg/ml). Successful transformants were selected by their EcoRI digestion pattern and were verified by dye-terminator sequencing (Lark Technologies). eGFP was cloned into the pENTR™ vector and selected as above. Both vectors were combined with the Lenti6/R4/R2/V5-DEST vector and incubated with LR Clonase™ Plus to produce an expression clone by homologous recombination. Expression constructs were transformed into One shot® Stbl3™ chemically-competent *E.coli*, selected with 100 µg/ml ampicillin and purified using a Qiagen endofree™ Maxi-prep column. UPK2-eGFP lentivirus produced from 293FT packaging cells as per the ViraPower Lentiviral Expression Systems protocol (Invitrogen) was filtered through a 0.45 µm filter and used immediately to transduce NHU cells in the presence of 6 µg/ml polybrene. Following selection of stable transductants with 1 µg/ml blasticidin, eGFP fluorescence was used as a correlate of UPK2 expression in NHU cells following induction of differentiation.

### MTT assay

The relative biomass of cultures was determined using a methyl thiazolyldiphenyl tetrazolium bromide (MTT) assay to assess mitochondrial dehydrogenase activity. NHU cells were seeded in 96-well plates at 2.5×10^3^ cells/well. One plate was left untreated to establish initial cell viability at T = 0. The remaining plates were incubated with inhibitors or recombinant growth factors in 6 replicates. To assay, wells were incubated with 200 µl MTT (0.5 mg/ml) at 37°C for 4 hours and the formazan crystals were dissolved in 200 µl DMSO and measured spectrophotometrically.

### Time lapse microscopy for scratch/migration analysis

NHU cells were seeded at 2.5×10^5^ cells/well in 24 well plates in KSFMc in the presence or absence of 5% ABS/2 mM CaCl_2_ and grown to confluence where cultures were contact-inhibited and out of cell cycle [18]. Media were replenished every 2–3 days. Cultures were pre-treated with TGFβ and/or inhibitors for 3 hours and then scratched once using a P100 pipette tip to generate a 500 µm wound, washed in KSFMc to remove cell debris and replenished with fresh medium containing appropriate treatment. Cultures were observed by differential interference contrast videomicroscopy (Olympus IX81 microscope) in an environmental chamber with an automated mechanical stage. Individual images were captured digitally every 10 minutes for 20 hours and were analysed by measuring the wound area and expressing it as the percentage healed compared to the original area of the wound in replicate (n = 3) cultures.

### Affymetrix Data

Following RNA extraction, mRNA was converted to cDNA and then to biotin-labelled cRNA before hybridising to Affymetrix™ GeneChip Human Genome U133 Plus 2.0 (HG-U133 Plus 2.0) arrays. The array chips were washed and scanned at 560 nm using an Affymetrix GeneChip Scanner.

The MAS5 Algorithm was used for background correction, normalisation and probe summarisation using ArrayAssist software (Affymetrix). Fold-change data were generated by comparing the non-differentiated proliferating control against the day 6 differentiated cultures. Changes to specific gene targets were validated on the target RNA and on RNA generated from independent NHU cell lines by real-time PCR.

### Real-Time PCR analysis

1 µg DNA-free RNA was reverse-transcribed using 50 ng of random hexamer primers and Invitrogen's Superscript™ II first-strand cDNA synthesis kit according to the manufacturer's protocol. Reverse transcriptase-negative and no template controls were included in all experiments and GAPDH was used as the normalisation transcript. Quantification of transcript expression was conducted on 10% of the cDNA reaction by real-time PCR using SYBR-green or TaqMan reagents (Applied Biosystems).

For relative quantification of transcript expression, template cDNA was mixed with SYBR-green PCR Master Mix and 400 nM of each forward and reverse target gene primer (Table 1) and analysed on an ABI Prism® 7300 Sequence Detection System. The thermal profile was: 2 minute hold at 50°C, incubation at 95°C (10 min), followed by 40 cycles of denaturation at 95°C (15 sec) and elongation at 72°C (1 min). Dissociation curves were performed to confirm the presence of a single amplification product and the absence of primer dimers for each primer set. Assay efficiency was validated using the C_T_ slope method prior to use and both test and endogenous assays were shown to be of equal efficiency. SYBR-green results were expressed as relative quantification (RQ) values (Applied Biosystems).

**Table 1:** Primers for SYBR-green RTqPCR.

Gene	Reference Sequence	Primer Sequence
**SMAD3**	NM_005902	**TCCAATTCGGAGCGCTTCT**
		ACTGCTGCATTCCTGTTGACA
**TGFβRI**	NM_004612	**AGCGGTCTTGCCCATCTTC**
		CTATGAGCAATGGCTGGCTTT
**TGFβRII**	NM_003242	**TGTCTGTGGATGACCTGGCTAA**
		TTCTGGAGCCATGTATCTTGCA
**SMURF2**	NM_022739	**CGTGGAGAAGAAGGCCTTGA**
		CATGTGACAAGAGATACAACCATTCC

Sequences shown are 5′-3′ with forward primer in bold.

For TaqMan, 5 µl template cDNA was mixed with 12.5 µl of TaqMan 2× Universal Master Mix (Applied Biosystems), 300 nM of each forward and reverse UPK2 primers, 200 nM of the UPK2 probe (Vic fluorophore), 200 nM each of GAPDH forward and reverse primers and 150 nM of the GAPDH probe (Fam fluorophore) as described [19]. Serial dilutions of human genomic DNA (100 - 0.16 ng) were used to generate standard curves. Normalised values of UPK2 mRNA were obtained by dividing the UPK2 amount by the GAPDH endogenous reference amount.

### Western blotting

Cultures were lysed into reducing electrophoresis sample buffer containing 0.2% (v/v) protease inhibitors (Cocktail Set III, Calbiochem) and assayed with a Coomassie protein assay reagent kit (Pierce). Proteins were resolved by electrophoresis on 1 mm 4–12% Bis-Tris NuPAGE pre-cast polyacrylamide gels (Novex, Invitrogen). 20–25 µg of sample was loaded alongside 5 µl of All-Blue pre-stained markers (BioRad), electrophoresed in MOPS or MES running buffer and electrotransferred to Immobilon PVDF transfer membrane (Millipore). Membranes were blocked in Odyssey™ blocking buffer (LI-COR systems) for 1 hour, probed with primary antibody then secondary antibodies before being visualised by infrared scanning followed by analysis on an Odyssey LI-COR infrared scanner. β-actin was used as a loading control to normalise band density to protein loading. Blots were stripped for reprobing in Western Blot Recycling Kit reagent (Autogen Bioclear) for 30 minutes at ambient temperature.

### Immunocytochemistry

NHU cells were grown on Teflon®-coated 12-well glass slides and fixed in a 1∶1 mixture of methanol∶acetone and air-dried, or fixed in 10% (v/v) formalin for 15 minutes and washed. Formalin-fixed slides were permeabilised in 0.1% (w/v) Triton-X. Primary antibody was applied overnight at 4°C, followed by incubation with secondary antibody conjugated to Alexa 488 or Alexa 594. Nuclei were visualised by staining with 0.1 µg/ml Hoechst 33258. Slides were examined under epifluorescence illumination using an Olympus BX60 microscope.

### Electrophysiology

Electrophysiological measurements were performed as described [11]. NHU cell cultures were grown on 4 µm pore Snapwell chambers (Corning Incorporated) for seven days in triplicate. Electrophysiological properties were measured using a VCC MC2 multi-channel voltage-current clamp (Physiologic Instruments Inc) and U-2500 vertical modified Ussing chambers (Warner Instruments) designed to fit the Snapwell inserts. The spontaneous potential difference (V, measured in mV) and short circuit current (I, measured in µA) across the urothelial cell layer were measured using Ag-AgCl microelectrodes connected to the voltage-current clamp. The transepithelial electrical resistance (TER) was calculated using Ohm's law (I = V/R) and adjusted for the surface area of the membrane (1.13 cm^2^) to give the TER in Ω.cm^2^.

### Statistics

Parametric statistics (mean and SD(n-1)) were used throughout for descriptive purposes and tests of significance were by means of a two-tailed t-test.

## Results

### Urothelial cytodifferentiation and associated gene changes

**Figure 1:**
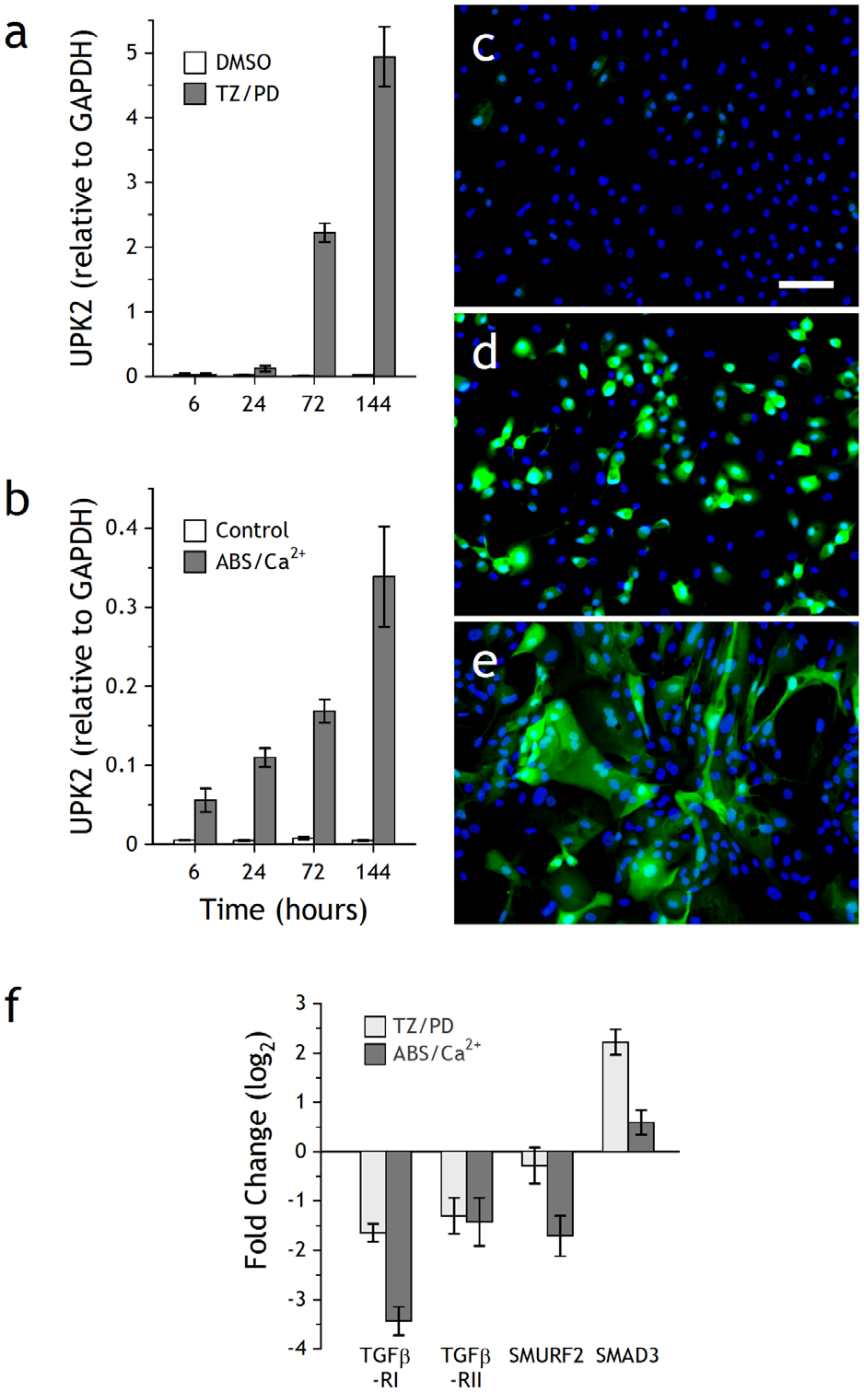
Analysis of differentiating NHU cells. NHU cells were differentiated by (a) TZ/PD or (b) ABS/Ca^2+^ protocols, as described in Materials and Methods, for the times indicated. Following RNA extraction, cDNA was generated and absolute quantitative PCR for UPK2 was performed. The VIC-labelled UKP2 product was normalised against the internal control (FAM-labelled GAPDH). Bars represent mean ± SD of triplicate PCR determinations. NHU cells transduced with UPK2-eGFP lentivirus were (c) left untreated, or differentiated by (d) TZ/PD or (e) ABS/Ca^2+^ protocols and assessed by epifluorescence; 6 day timepoint shown. Scale bar 80 µm. To verify changes in TGFβ target gene expression during differentiation, cultures from an independent NHU cell line were induced to differentiate with TZ/PD or ABS/Ca^2+^ for 72 h (f). RTqPCR using comparative quantification by cycle threshold (C_T_) with SYBRgreen was performed in triplicate and normalised against the internal control (GAPDH). Data show TGFβ pathway-associated gene expression in response to treatment with TZ/PD or ABS/Ca^2+^ versus vehicle control. Bars represent mean ± SD.

Expression of urothelium differentiation-restricted uroplakin 2 (UPK2) transcript was used as a marker to monitor the differentiation status of NHU cell cultures. Whereas there was minimal UPK2 expression in control cultures maintained in standard growth medium, both differentiation-inducing protocols (TZ/PD and ABS/Ca^2+^) resulted in time-dependent increases in UPK2 transcript (Figs. 1A–B). NHU cultures stably transduced with UPK2-eGFP lentivirus contained few eGFP-expressing cells even at confluence (Fig. 1C). Following induction of differentiation, a majority of cells became fluorescent within 6 days. Cells differentiated pharmacologically by TZ/PD remained discrete and rounded, whereas those differentiated by ABS/Ca^2+^ formed integrated, overlying cell sheets (Figs. 1D–E). To identify gene expression changes common to both differentiation strategies, gene arrays were performed on cRNA derived from parallel cultures harvested at 6 days post induction of differentiation. The array data for the two differentiation-inducing protocols was validated by examining changes in expression of marker genes associated with differentiated urothelium, including UPK2 (Table S1).

A pair-wise comparison of the arrays against a non-differentiated control was used to identify genes that showed significant (p<0.01) changes in expression based on a log_2_-fold change in signal intensity. Using the TZ/PD protocol, the expression of 2511 genes was significantly increased and 2926 showed a decrease. With ABS/Ca^2+^, 2326 genes were increased and 1984 decreased. 2116 gene changes were common to both protocols.

The method-independent subset of gene changes common to both differentiation-inducing protocols was assessed by functional ontology within the Ingenuity™ Systems software suite. This indicated gene expression changes implicated in programs of cellular growth/proliferation, migration, death, and cell-cell signaling. Within the growth factor signalling category, TGFβ, IGF-1, HGF and EGF emerged as the top four modified pathways, with 8/83 (9.6%), 7/100 (7.0%), 7/103 (6.8%) and 3/49 (6.1%) gene changes for each pathway, respectively.

### Differentiation-associated changes in the TGFβ pathway

Pathway analysis with Ingenuity software was used to infer (at the transcript level) which components of the TGFβR pathway components were expressed and/or modified by differentiation. Present in both proliferative and differentiated cultures were expressed all the necessary pathway components for the canonical TGFβR/Activin/SMAD pathway, whereas components of the BMP/Nodal-related SMAD signalling pathway were either absent or showed no change in expression following differentiation (Table 2; a full profile of TGFβ type I and II receptor expression is shown in Supplementary Table S2).

**Table 2:** Affymetrix™ genechip analysis in differentiated versus proliferative NHU cells.

		ABS/Ca^2+^	TZ/PD
**Ligand**	TGFβ1	−3.62	nc
	TGFβ2	+5.30	nc
	TGFβ3	A	A
**Receptor**	TGFβRI	−1.69	−1.53
	TGFβRII	−7.06	−2.29
**R-SMAD**	SMAD1	nc	nc
	SMAD2	nc	nc
	SMAD3	+5.06	+3.79
	SMAD5	nc	nc
	SMAD9	A	A
**Co-SMAD**	SMAD4	nc	nc
**I-SMAD**	SMAD6	A	A
	SMAD7	−1.44	−2.02
**SMURF**	SMURF1	nc	nc
	SMURF2	−2.77	−4.27

NHU cells were differentiated with TZ/PD or ABS/Ca^2+^ and harvested after 6 days. Biotin-labelled cRNA generated from control (proliferating) and 6 day differentiated cultures was hybridised to U133 genechips.

Numbers represent fold change; + and − indicate increased and decreased expression, respectively; nc = no change and A = absent.

From the arrays, SMAD3 was up-regulated >3-fold in ABS/Ca^2+^ and >5-fold in TZ/PD differentiated cell cultures. Although there was no change in SMAD2 transcript expression, there was a decrease in SMURF2, a SMAD ubiquitination regulatory factor responsible for targeting SMAD2 for degradation. There was downregulation of transcripts for inhibitory SMAD7 and for TGFβ-RI and TGFβ-RII. These trends were confirmed by comparative RTqPCR on samples generated from an independent cell line at 72 hours post induction of differentiation (Fig. 1F).

**Figure 2:**
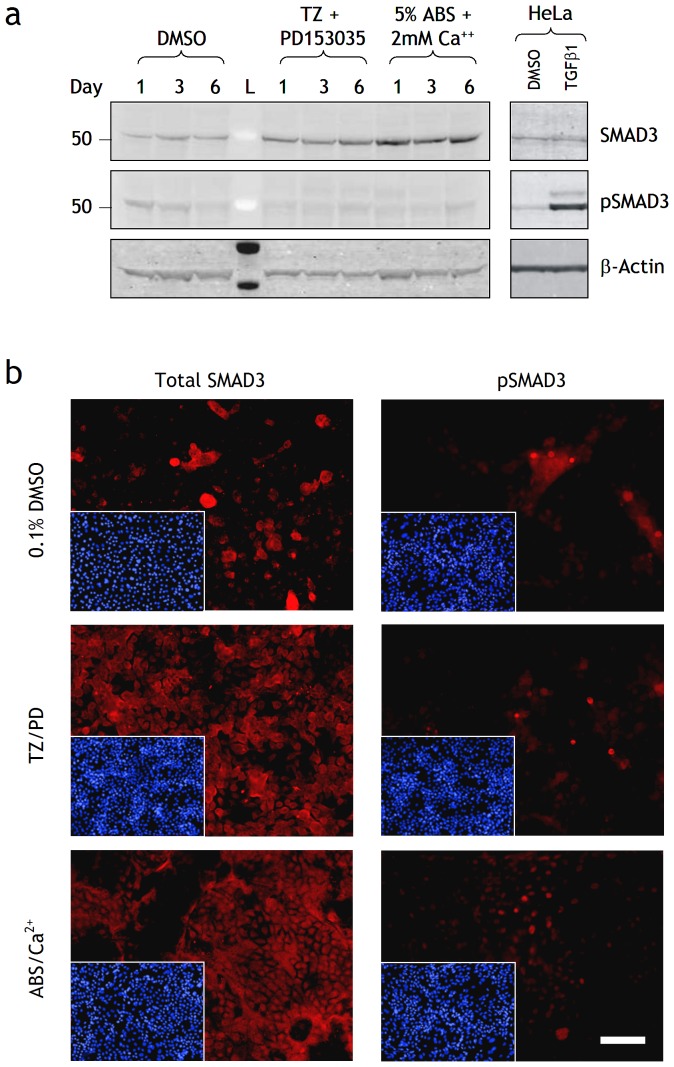
Effect of differentiation on SMAD3 and pSMAD3 protein expression in NHU cells. (a) Protein lysates were prepared from NHU cells treated with 0.1% DMSO (control), TZ/PD or ABS/Ca^2+^ for 1, 3 and 6 days. Cell extracts (25 µg) were resolved on 4–12% Bis-Tris polyacrylamide gels and transferred onto PDVF membranes. Membranes were incubated with titrated primary antibodies for 16 h at 4°C to SMAD3 and pSMAD3, as indicated. Bound antibody was detected with Alexa Fluor® 680 and LI-COR IRDye™ 800 conjugated secondary antibodies and visualised using the Odyssey™ Imaging System. β-actin was used as an internal loading control. HeLa cells treated with TGFβ1 (2 ng/ml) for 24 h was used as a positive control. L is the molecular size ladder. (b) NHU cells were seeded at 500 cells/cm^2^ onto glass slides, allowed to adhere and treated with or without TZ/PD or ABS/Ca^2+^ for 6 days and fixed in formalin. Media were replaced every 3 days with fresh treatments. Indirect immunofluorescence was performed with anti-SMAD3 and anti-pSMAD 3 antibodies as indicated and detected with Alexa 594-conjugated secondary antibodies. Picture inserts show the respective Hoescht 33258 nuclear stain. Scale bar 90 µm.

To determine whether changes in SMAD3 transcript translated to changes in protein expression, differentiating NHU cell cultures were analysed by immunoblotting (Fig. 2A). Total SMAD3 protein was increased following both differentiation treatments. The relative amount of phosphorylated SMAD3 (pSMAD3) decreased in control cells from days 1–6 as cells reached confluence. Differentiated cells expressed very low amounts of pSMAD3 indicating that although basal SMAD3 expression increased, actual activity based on phosphorylation decreased, possibly as a reflection of other changes to the pathway (Table 2). These findings were confirmed by immunofluorescence (Fig. 2B): SMAD3 immunolabelling was most intense in differentiated cells, but pSMAD3 labelling was low or absent in the majority of cells in all culture conditions, with intense nuclear labelling observed in occasional cells only.

### Effect of TGFβ on NHU cell morphology and growth

**Figure 3:**
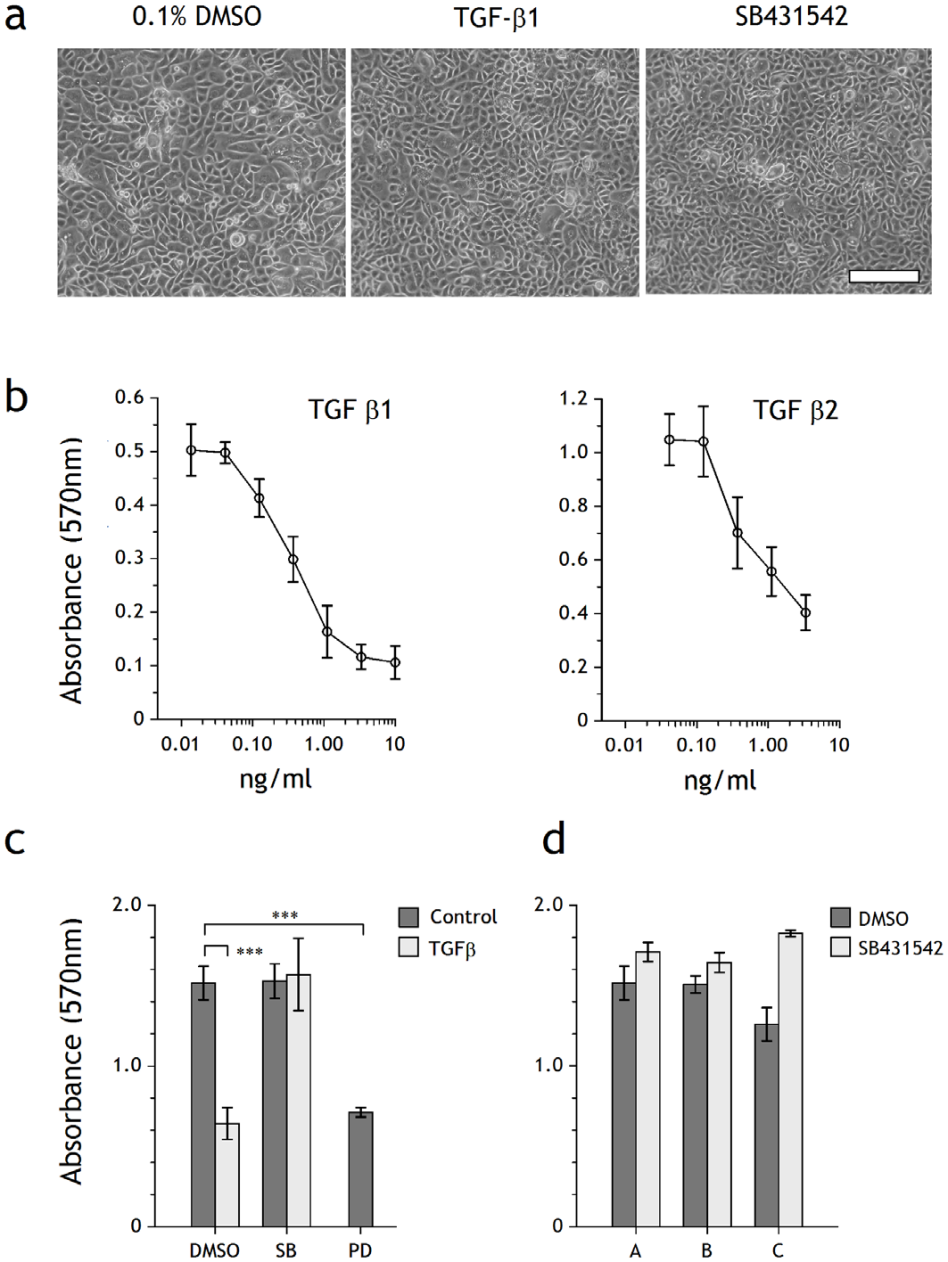
Influence of TGFβ and TGFβ inhibitors on morphology and proliferation of NHU cell cultures. (a) Phase-contrast micrographs of NHU cell cultures at confluence following growth for 6 days in the presence of 0.1% DMSO, TGFβ1 (2 ng/ml) or SB431542 (3 µM). Scale bar: 250 µm. (b) MTT assay showing dose-response curves of viable biomass from non-differentiated (proliferative) NHU cells treated for 4 days with TGFβ1 and TGFβ2. Bars represent mean ± SD of six replicates. (c) NHU cells were treated with the inhibitors SB431542 (10 µM) or PD153035 (1 µM) in the presence or absence of TGFβ1 (2 ng/ml) for 4 days and viable biomass was assessed by MTT assay. Bars represent means ± SD of six replicate wells. Statistical analysis was by means of a 2-tailed t-test, *** = p<0.0001. (d) NHU cells from three independent cell lines (A, B, C) were cultured in the presence or absence of SB431542 (3 µM) for 4 days before growth was assessed by MTT assay. Bars represent means ± SD of six replicate wells.

Neither exogenous TGFβ ligands, nor specific inhibition of TGFβRI tyrosine kinase with SB431542 had any effect on the morphology of NHU cells in culture (Fig. 3A).

MTT biomass assays performed over 6 days showed that TGFβ1 and TGFβ2 both induced dose-dependent growth inhibition of non-differentiated (proliferative) NHU cells, with IC_50_ values calculated as 0.2–0.4 ng/ml for TGFβ1 and 0.3–0.5 ng/ml for TGFβ2, respectively (Fig. 3B). Subsequent experiments were performed with 2 ng/ml TGFβ1, representing >80% (EC_80_) of the maximal response, which was equivalent to the inhibition induced by 1 µM PD153035, an EGFR-specific tyrosine kinase inhibitor included as positive control (Fig. 3C). 1–10 µM SB431542 abrogated the growth inhibitory action of TGFβ1 and, when used in the absence of exogenous TGFβ1, had a small but reproducible positive effect on culture biomass (Fig. 3D).

**Figure 4:**
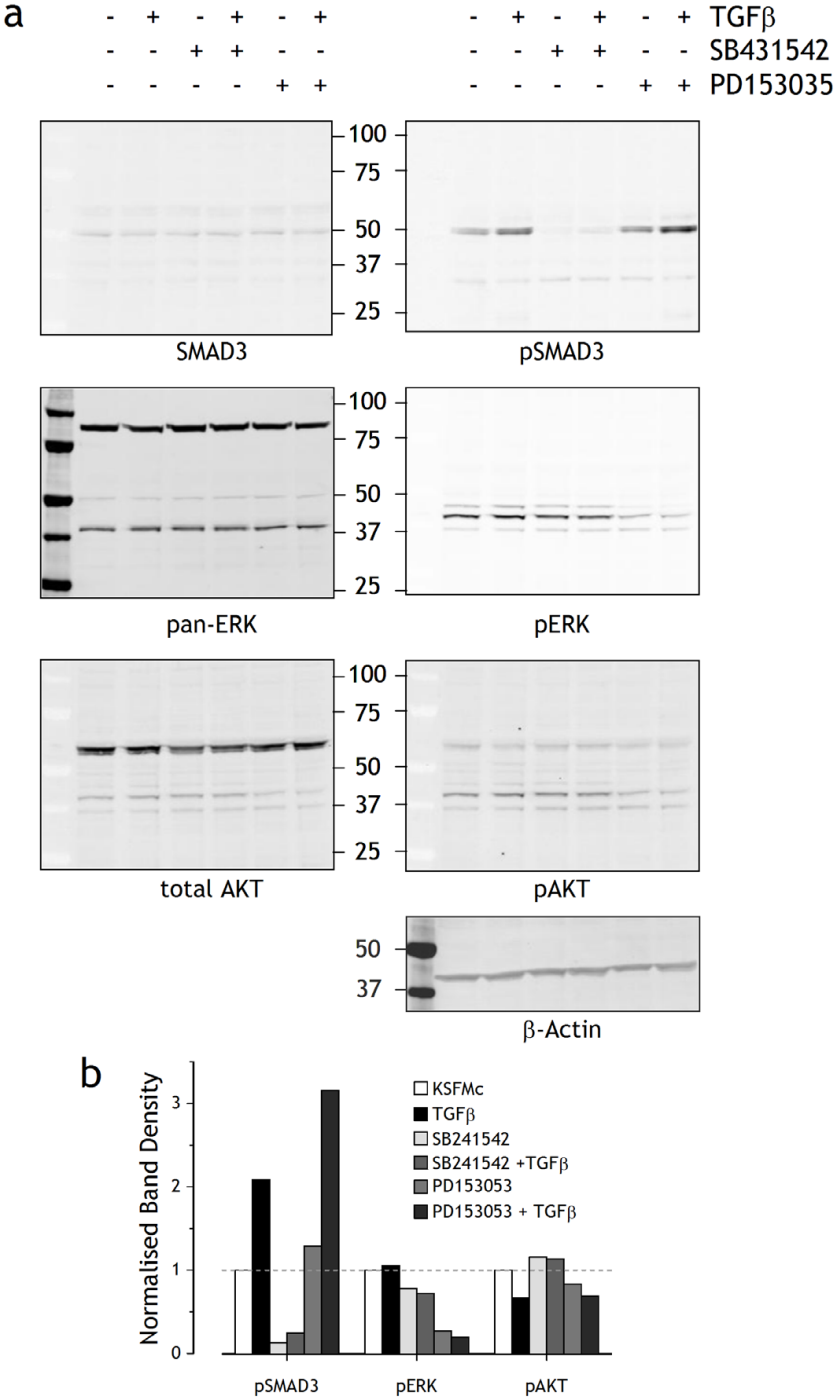
Immunoblot analysis of NHU cell response to TGFβ signalling. (a) Sub-confluent NHU cells were treated with or without PD153035 (1 µM), TGFβ (2 ng/ml) or SB431542 (3 µM) for 24 h before preparation of cell lysates. Cell extracts (15 µg) were analysed by Western blot analysis as described in Materials and Methods. β-actin was used as an internal loading control. (b) Gel band densities of the Western blots in (E) using the LI-COR Odyssey™ Imaging System and normalised to β-actin to show the changes in phosphorylated protein. The ratio of phosphorylated∶total protein in the control was taken as 1.0, as indicated by the dashed line.

SMAD3 was phosphorylated in NHU cells stimulated with TGFβ1 for 24 hours (Fig. 4). SB431542 inhibited TGFβ1-induced SMAD3 phosphorylation and abrogated the small basal expression of pSMAD3 seen in control cultures without exogenous TGFβ, indicating autocrine activity (Fig. 4), as the growth medium supplements contained minimal TGFβ activity and did not account for the baseline activity (Fig. S1). Modulation of TGFβ signalling with exogenous TGFβ ligand or SB431542 had small reciprocal effects on ERK and AKT signalling (Fig. 4). Inhibition of EGFR activity with PD153035 had little direct effect on SMAD3 activation, but enhanced the effect of TGFβ on pSMAD3 by >50%. This suggested cross-talk between the EGFR/TGFβR pathways, but the consequence of this for cell phenotype was not assessed in this study.

### Effect of TGFβ1 on NHU cytodifferentiation

**Figure 5:**
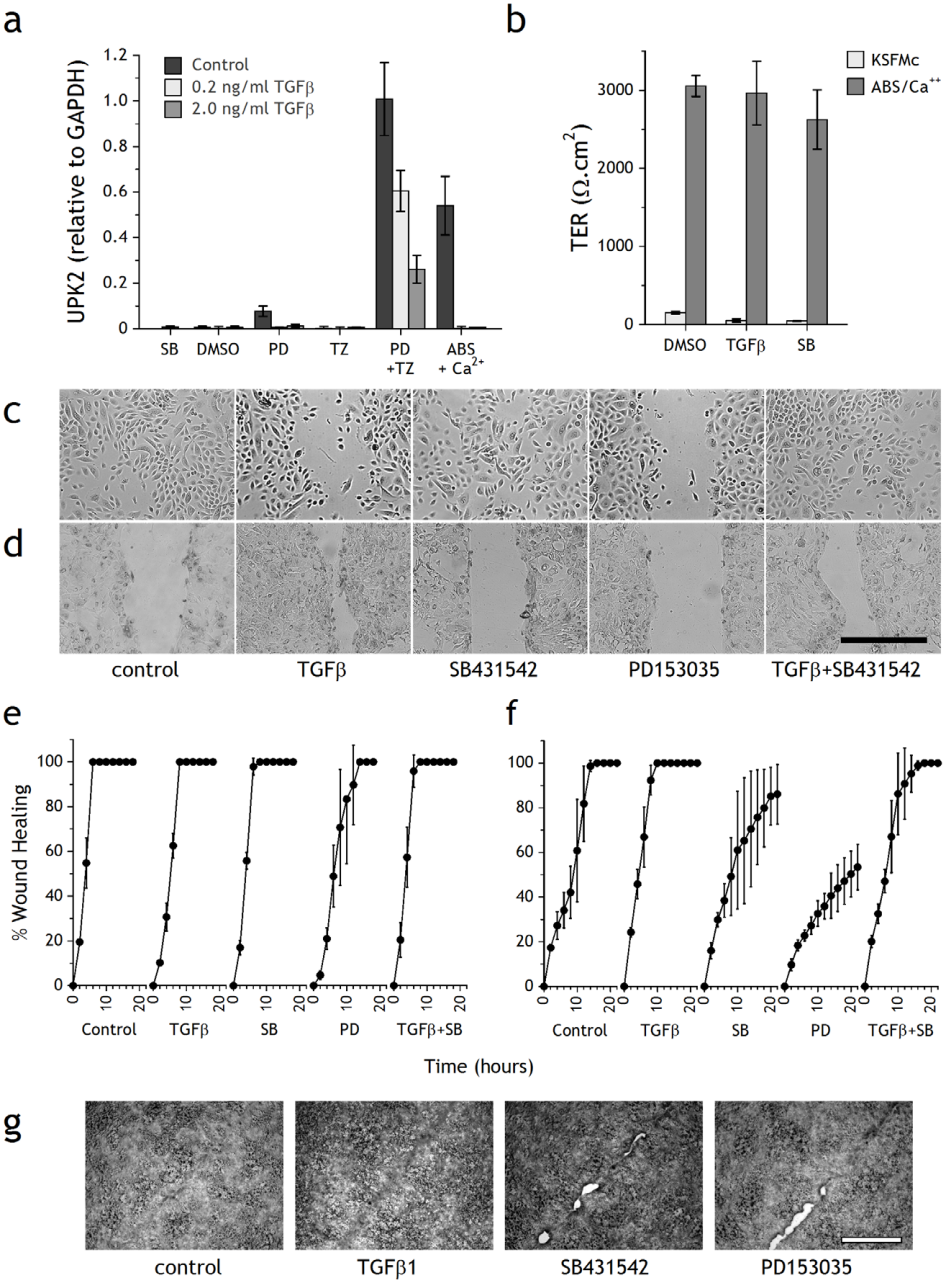
Effects of TGFβ on differentiation, barrier function and scratch wound repair in NHU cell cultures. (a) NHU cells were differentiated with or without TZ/PD or ABS/Ca^2+^ in the presence or absence of TGFβ; UPK2 expression was assessed by RTqPCR after 4 days and normalised against GAPDH. Controls included SB431542, vehicle (0.1% DMSO), PD153035 and TZ alone. Bars represent means ± SD of triplicate PCR determinations. (b) Barrier function was assessed by TER measurement after 7 days culture of NHU cells seeded at 0.5×10^6^ cells/cm^2^ onto SnapWell™ membranes and treated with or without ABS/Ca^2+^ in the presence of DMSO (0.1%), TGFβ1 (2 ng/ml) or SB431542 (3 µM). Bars represent mean ± SD of 3 independent replicate cultures. (c–g) After 7 days pre-culture with or without ABS/Ca^2+^ to induce differentiation, confluent cultures were incubated with DMSO (0.1%, vehicle control), TGFβ1 (2 ng/ml), SB431542 (10 µM) or PD153035 (1 µM) for 3 h and then scratch-wounded. Cultures were maintained in respective treatments in an environmental chamber and images were taken every 10 minutes by time-lapse microscopy until the wounds healed. The experiment was repeated on 3 independent NHU cell lines with the same results. Differential interference contrast micrographs are shown for (c) non-differentiated (6 h) and (d) differentiated cultures (8 h) post-wounding; scale bar 500 µm. The time-course of wound repair was quantified from timelapse micrographs at 2 hour intervals and expressed against the original wound as the percentage healed for confluent (e) non-differentiated and (f) differentiated cultures. Points represent means of triplicate cultures ± SD. Phase contrast micrographs (g) of repaired scratch wounds in differentiated NHU cell cultures after 16 h in the presence of DMSO (0.1%), TGFβ1 (2 ng/ml), PD153035 (1 µM) or SB431542 (3 µM). Scale bar: 500 µm.

The changes observed in TGFβ-associated transcript and protein expression suggested that modulation of TGFβ/activin signalling was involved in the differentiation of human urothelial cells. To test this hypothesis, the effect of exogenous TGFβ1 was assessed on expression of UPK2 transcript by quantitative RT-PCR (Fig. 5A). As a positive control, NHU cultures were induced to differentiate using the TZ/PD and ABS/Ca^2+^ protocols. TGFβ1 alone or in combination with PD153035 or TZ did not induce UPK2 expression, showing that TGFβ1 alone does not induce or promote differentiation in NHU cell cultures. Inclusion of TGFβ1 into the TZ/PD protocol actually reduced expression of UPK2 transcript, implying an inhibitory effect on differentiation by that exogenous TGFβ1 (Fig. 5A). Inclusion of SB431542 to inhibit TGFβ signalling did not significantly affect UPK2 transcript expression (not shown).

Transepithelial electrical resistance can be used as a functional measure of urothelial differentiation and barrier formation. NHU cell cultures grown in standard growth conditions in KFSMc medium did not develop a tight barrier, with TER measurements of <100 Ω.cm^2^, irrespective of whether or not they were cultured with 2 ng/ml TGFβ1 or 3 µM SB431542. The presence of exogenous TGFβ1 or SB431542 during NHU differentiation induced by the ABS/Ca^2+^ protocol did not significantly affect barrier function, with TER values in the region of 2640–3050 Ω.cm^2^ (Fig. 5B).

### Effect of TGFβ1 on urothelial scratch-wound repair

To investigate whether TGFβ1 signalling had a specific role in regenerative repair, confluent, contact-inhibited cultures of non-differentiated and ABS/Ca^2+^ differentiated NHU cells were scratch-wounded in the presence of 2 ng/ml TGFβ1 and/or 3 µM SB431542 or 1 µM PD153035, and the repair was monitored by time-lapse microscopy. In non-differentiated cultures, 500 µm scratch wounds repaired within 6 hours in both control and SB431542-treated cells, but repair was retarded by exogenous TGFβ1, or by inhibiting EGFR with PD153035 (Figs. 5C and 5E).

By contrast, in differentiated NHU cell cultures (Fig. 5D), the repair of 500 µm scratch wounds was accelerated when exogenous TGFβ1 was present and was blocked back to control values by SB431542. Repair was inhibited by PD153035 or by SB431542 alone. Measurement of the time taken to achieve full repair showed that TGFβ-treated and control cultures had healed by 10 and 14 hours, respectively, whereas wounds treated with SB431542 or PD153035 did not heal over the time course of the experiment (Fig. 5F). In the presence of exogenous TGFβ1, the repaired region appeared as a pronounced “scar” (Fig. 5G). By contrast, SB431542 or PD153035 compromised the quality of the final repair, resulting in persistent unhealed patches along the wound (Fig. 5G). Combined treatment with PD153035 and SB431542 did not have an additive effect on wound repair, suggesting they act through a common pathway (not shown).

**Figure 6:**
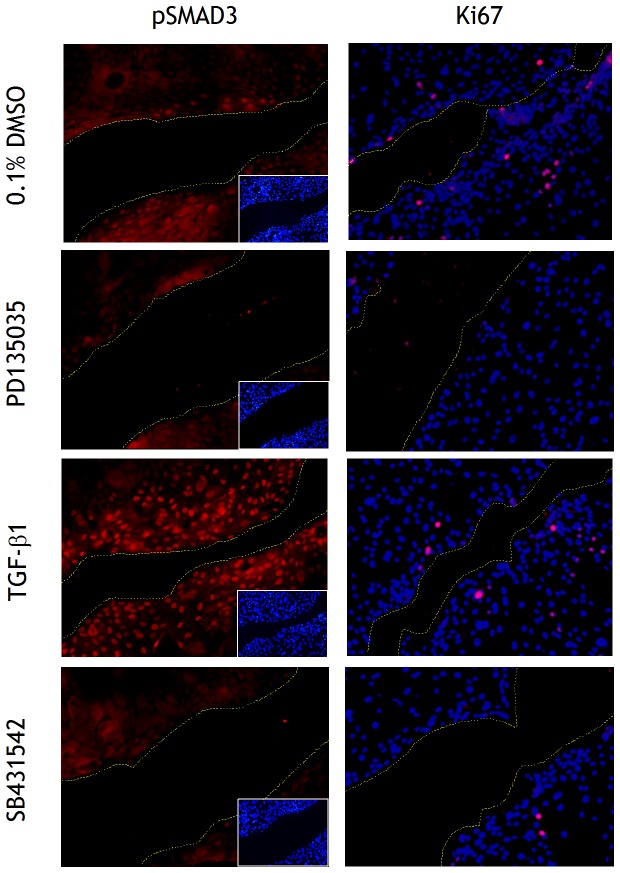
Localisation of phospho-SMAD3 in scratch-wounded differentiated NHU cell cultures. NHU cells were induced to differentiate by 7 day culture with ABS/Ca^2+^ prior to treatment with DMSO (0.1%, vehicle control), TGFβ1 (2 ng/ml), SB431542 (3 µM) or PD153035 (1 µM) for 3 hours and then scratched. Cells were maintained in treatments for 8 hours post-wounding and formalin fixed before immunofluorescence labelling as described in the Materials and Methods. Picture inserts or merged images show the respective Hoechst 33258 nuclear stain. Yellow lines indicate wound margins. Scale bar: 100 µm.

pSMAD3 immunolabelling was investigated in ABS/Ca^2+^-differentiated cultures following scratch-wounding in the presence of 2 ng/ml TGFβ1, 3 µM SB431542 or 1 µM PD153035 (Fig. 6). In control cultures, pSMAD3 was only detected in a few cells immediately along the wound edge and a few cells back. This was weaker in intensity, but similar in distribution to the pattern of pSMAD3 labelling observed in TGFβ1-treated cultures, where intense nuclear labelling was present in the 5–10 cells adjacent to the wound edge (Fig. 6). Cultures treated with either SB431542 or PD153035 showed limited pSMAD3 labelling along the wound edge, but this was very much reduced relative to controls.

A very few cells surrounding the wound edge were in the mitotic cycle in control and TGFβ1-treated cultures, as judged by Ki67 immunofluorescence. There was no evidence of Ki67 labelling in cultures treated with SB431542. This suggests that migration rather than proliferation was the predominant event contributing to closure of the scratch wound (Fig. 6).

## Discussion

TGFβ is a pleiotropic cytokine implicated in tissue development, repair, remodelling and pathogenesis. The diversity of its effects is presumed to be dependent on both the target cell type and signalling context, including ligand sequestration and the modulation of ligand release. Interpretation of TGFβ signalling is further complicated by biphasic effects, where responses induced at low concentrations are different or absent at higher concentrations of agonist, or which, in combination with a second stimulus, can invoke different responses [20]. Thus, dissection of the precise relationship between TGFβ signalling and cell/tissue response is, by its very nature, confounded by the complexity of the biological system studied. This study of a normal human in vitro system that represents both regenerative and differentiated states has provided novel insights. We have shown that TGFβ signalling is fundamentally different in proliferating and differentiated urothelial cell cultures, respectively, both in terms of the nature of the activating signal (paracrine versus autocrine) and the output response (growth inhibition versus wound-healing). The findings revealed that urothelial cytodifferentiation induces major changes in the functional programming of the canonical pathway and this itself has important implications for pathway modulation in pathological states. Critically, our study demonstrates that this pathway reprogramming is secondary to the differentiation process itself, but contributes to a fundamental property of the mature tissue in its potential for self-regeneration.

It is axiomatic that the activity of a signal transduction pathway is modulated through activation/inhibition of the components rather than direct changes in their expression. However, our transcriptome and ontology analysis revealed that the TGFβ/TGFβR/Activin/SMAD signalling pathway is a major target for transcriptional reprogramming during urothelial differentiation. Confidence that this is a fundamental, differentiation-associated change is supported by the observation that two independent differentiation-inducing protocols – one initiating the terminal differentiation transcriptional programme in individual cells and the other enabling differentiated tissue development - both induced common changes in the TGFβR signal transduction pathway. This was a wholly unexpected observation and suggests a paradigm shift in pathway function. Whereas there was reduced expression of the two TGFβ receptors in differentiated cells, this was countered by an upregulation of both SMAD3 effector and SMURF2, a SMAD ubiquitination regulatory factor responsible for targeting SMAD2 for degradation. In addition, there was downregulation of SMAD7, which negatively regulates TGFβRI through interactions with SMURF2. The reduced receptor expression in association with an upregulation of effector and downregulation of inhibitory components of the pathway indicates a shift in the threshold for pathway activation and the potential for a more vigorous response once activation has been triggered.

TGFβ has long been associated with growth regulation and differentiation during tissue development and repair, and in some systems, it has been reported that TGFβ induced differentiation as a corollary of its growth inhibitory effects [21], [22]. However, far from inducing differentiation, we found that exogenous TGFβ had an inhibitory effect. A role for TGFβ signalling in urothelial cytodifferentiation has been previously suggested [23], [24] and these reports are consistent with our interpretation of this study – namely that the TGFβ signalling machinery is altered as a consequence, rather than function of urothelial differentiation.

Because TGFβR signalling had no apparent role in the differentiation process itself, we examined its contribution to wound repair in differentiated urothelial cell sheets. These experiments revealed that repair was inhibited by the blocking of TGFβR-specific tyrosine kinase in absence of exogenous TGFβ and also that exogenous TGFβ induced hypertrophic scarring. The former observation is indicative of autocrine TGFβR signalling, which is further supported by the immunolocalisation evidence showing pSMAD3 activation in proximity to the wound edge. Thus, the machinery for autocrine activation of the SMAD3-mediated TGFβR pathway is established during differentiation, but activation occurs only in response to a trigger, such as wounding.

The urothelium functions as a urinary barrier, for which the ability to repair (proliferation) and to provide a physical barrier (differentiation) are both critical but conflicting attributes. In its normal state, human urothelium is mitotically-quiescent, but individual cells retain a high proliferative capacity. Whereas there is evidence, particularly in the mouse, that paracrine signalling from other tissue compartments such as the stroma may play a role in driving urothelial repair [4], there is also evidence, shown here and elsewhere [5] that at least in man, the urothelium functions autonomously through autocrine signalling to regulate self-repair.

A pre-requisite for epithelial tissue wound repair is the release of cells from the epithelial community to assume a migratory, “wound-healing” phenotype. This is achieved by dissolution of tight junctions which, with ERK-mediated dissolution of adherens junctions [25] is part of the EMT phenomenon mediated by TGFβ. In non-differentiated NHU cell culture, the Ca^2+^ concentration (0.09 mM) is below the threshold required for E-cadherin homodimerisation, which constrains the formation of adherens and tight junctions. Thus, NHU cells assume an EMT-like phenotype, as evidenced by migratory behaviour, deposition of fibronectin and expression of the cognate α5β1 integrin receptor [26]. In such conditions, proliferating NHU cells responded to exogenous TGFβ by growth arrest and by inhibition of migration in scratch wound repair. This cytostatic response is in keeping with the recognised role of TGFβ as a tumour suppressor that promotes G1-cell cycle arrest through induction of the cyclin-dependent kinase inhibitors [27], [28].

Thus we provide a unifying hypothesis to explain the apparent paradoxical effects of TGFβR signalling on epithelial repair and differentiation in which the hardware and circuitry for autocrine activation of the SMAD3-mediated TGFβR pathway is established during differentiation, but activation occurs only in response to a trigger, such as wounding.

## Supporting Information

Figure S1 **Influence of medium supplements on the basal phosphorylation of SMAD3 in NHU cell cultures.** NHU cells were grown in KSFMc or KSFM with no supplements, with BPE alone (60 µg/ml) or EGF alone (6 µg/ml), all in the presence or absence of SB431542 (3 µM). KSFMc with TGF-β1 (2 ng/ml) was used as a positive control for pSMAD3 activation. Cell lysates (25 µg) were assessed by Western blot analysis as described in the Materials and Methods. β-actin was used as an internal loading control. Similar results were found with HeLa cells (not shown).
(DOCX)

Table S1 **Verification that differentiated NHU cell cultures used for Affymetrix arrays expressed established urothelial differentiation-associated genes.** Analysis of marker gene expression from arrays performed at 144 h post induction of differentiation by ABS/Ca^2+^ and TZ/PD protocols compared to the autologous 24 h non-differentiated control culture. Results expressed as log_2_ fold change. A minus denotes a reduction in expression. The panel of marker genes assessed were: ***PLK1***. Cell cycle/proliferation marker. **Cytokeratins**. Whereas KRT7 is expressed by all urothelial layers in situ and showed no change following differentiation; KRT13, a marker of transitional differentiation was upregulated and the KRT14 squamous differentiation marker was downregulated [14]. **Uroplakins**. Urothelial differentiation was accompanied by expression of uroplakin genes, which in human are restricted to the terminally-differentiated superficial urothelial cells [2], [13]. **Claudins**. Changes in tight junction composition accompany urothelial differentiation, including expression of claudin 4 [12].
(DOC)

Table S2 **Expression of TGFβ ligands and probes taken from analysis of gene chip data.** P = present; A = absent
(DOC)
