# Supplementary material for: Differentiation-Associated Reprogramming of the Transforming Growth Factor β Receptor Pathway Establishes the Circuitry for Epithelial Autocrine/Paracrine Repair
Source: PLoS One. 2012 Dec 19;7(12):e51404. doi: 10.1371/journal.pone.0051404 (PMC3526617; doi:10.1371/journal.pone.0051404)
Supplement: Figure S1 — Influence of medium supplements on the basal phosphorylation of SMAD3 in NHU cell cultures. NHU cells were grown in KSFMc or KSFM with no supplements, with BPE alone (60 µg/ml) or EGF alone (6 µg/ml), all in the presence or absence of SB431542 (3 µM). KSFMc with TGF-β1 (2 ng/ml) was used as a positive control for pSMAD3 activation. Cell lysates (25 µg) were assessed by Western blot analysis as described in the Materials and Methods. β-actin was used as an internal loading control. Similar results were found with HeLa cells (not shown). (DOCX) [file pone.0051404.s001.docx]

### Figure S1

|  |  |
| --- | --- |
|  |  |
