# Supplementary material for: Differentiation-Associated Reprogramming of the Transforming Growth Factor β Receptor Pathway Establishes the Circuitry for Epithelial Autocrine/Paracrine Repair
Source: PLoS One. 2012 Dec 19;7(12):e51404. doi: 10.1371/journal.pone.0051404 (PMC3526617; doi:10.1371/journal.pone.0051404)
Supplement: Table S1 — Verification that differentiated NHU cell cultures used for Affymetrix arrays expressed established urothelial differentiation-associated genes. Analysis of marker gene expression from arrays performed at 144 h post induction of differentiation by ABS/Ca2+ and TZ/PD protocols compared to the autologous 24 h non-differentiated control culture. Results expressed as log2 fold change. A minus denotes a reduction in expression. The panel of marker genes assessed were: PLK1. Cell cycle/proliferation marker. Cytokeratins. Whereas KRT7 is expressed by all urothelial layers in situ and showed no change following differentiation; KRT13, a marker of transitional differentiation was upregulated and the KRT14 squamous differentiation marker was downregulated [14]. Uroplakins. Urothelial differentiation was accompanied by expression of uroplakin genes, which in human are restricted to the terminally-differentiated superficial urothelial cells [2], [13]. Claudins. Changes in tight junction composition accompany urothelial differentiation, including expression of claudin 4 [12]. (DOC) [file pone.0051404.s002.doc]

## Table S1

#### Verification of differentiation by NHU cell cultures used for Affymetrix arrays.

| **Gene** | **ABS/Ca** | **TZ/PD** |
| --- | --- | --- |
| PLK1 | -3.78 | -2.38 |
| PCNA | 0.34 | 0.11 |
| KRT 7 | 1.24 | 2.09 |
| KRT13 | 2.80 | 2.85 |
| KRT 14 | -4.77 | -3.13 |
| UPK2 | 2.53 | 4.72 |
| UPK3a | 6.38 | 7.25 |
| CLDN4 | 3.57 | 4.42 |

Analysis of marker gene expression from arrays performed at 144h post induction of differentiation by ABS/Ca^2+^ and TZ/PD protocols compared to the 24h non-differentiated, proliferating NHU control culture. Results expressed as log_2_ fold change. A minus denotes a reduction in expression.

The panel of marker genes assessed were:
***PLK1***. Cell cycle/proliferation marker.
**Cytokeratins**. Whereas KRT7 is expressed by all urothelial layers in situ and showed no change following differentiation; KRT13, a marker of transitional differentiation was upregulated and the KRT14 squamous differentiation marker was downregulated [14].
**Uroplakins**. Urothelial differentiation was accompanied by expression of uroplakin genes, which in human are restricted to the terminally-differentiated superficial urothelial cells [2,13].
**Claudins**. Changes in tight junction composition accompany urothelial differentiation, including expression of claudin 4 [12].
