# Supplementary material for: Differentiation-Associated Reprogramming of the Transforming Growth Factor β Receptor Pathway Establishes the Circuitry for Epithelial Autocrine/Paracrine Repair
Source: PLoS One. 2012 Dec 19;7(12):e51404. doi: 10.1371/journal.pone.0051404 (PMC3526617; doi:10.1371/journal.pone.0051404)
Supplement: Table S2 — Expression of TGFβ ligands and probes taken from analysis of gene chip data. P = present; A = absent (DOC) [file pone.0051404.s003.doc]

## Table S2

#### Expression of TGFβ ligands and probes taken from analysis of gene chip data.

|  |  | **Proliferating** | **Differentiated** | |
| --- | --- | --- | --- | --- |
|  |  |  | **ABS/Ca^2+^** | **TZ/PD** |
| **TYPE II** |  |  |  |  |
| ACVR2A | activin A receptor, type IIA | P | P | P |
| ACVR2B | activin A receptor, type IIB | A | A | A |
| TGFBR1 | transforming growth factor β receptor I (activin A receptor type II-like kinase, 53kDa) | P | P | P |
| BMPR2 | bone morphogenetic protein receptor type II (serine/threonine kinase) | P | P | P |
| AMHR2 | anti-Mullerian hormone receptor type II | A | A | A |
|  |  |  |  |  |
| **TYPE I** |  |  |  |  |
| ACVR1B | activin A receptor, type IB | A | A | A |
| TGFBR2 | transforming growth factor β receptor II (70/80kDa) | P | P | P |
| ACVR1C | activin A receptor type IC | A | A | A |
| BMPR1A | bone morphogenetic protein receptor type IA | P | P | P |
| BMPR1B | bone morphogenetic protein receptor type IB | A | A | A |
| ACVRL1 | activin A receptor type II-like 1 | A | A | A |
| ACVR1 | activin A receptor type I | P | P | P |

P = present; A = absent
